# Improving patient specific quality assurance for image registration: clinical use case of target contouring for PET deformable image registration

**DOI:** 10.1007/s13246-025-01541-1

**Published:** 2025-05-14

**Authors:** Johnson Yuen, Joel Poder, Michael Jameson, Laurel Schmidt, Ryan Brown, Charlotte Atkinson, Shrikant Deshpande, Anna Ralston, Lois Holloway

**Affiliations:** 1 Cancer Care Centre, Department of Medical Physics, St. George Hospital, Kogarah, NSW Australia; 2 South Western Clinical School, University of New South Wales, Sydney, Australia; 3 Ingham Institute for Applied Medical Research, Sydney, Australia; 4 Centre for Medical Radiation Physics, University of Wollongong, Wollongong, NSW Australia; 5 GenesisCare St. Vincent’s Clinic, Sydney, NSW Australia; 6 Liverpool and Macarthur Cancer Therapy Centres, Liverpool, NSW Australia; 7 Queens Hospital, Romford, Essex UK

**Keywords:** Deformable image registration, Adaptive radiotherapy, Contour propagation, Quality management, Multi-modality image registration, PET, Target delineation

## Abstract

Deformable image registration (DIR) has proven to be an invaluable tool to maximize the clinical benefits of multimodality imaging in radiation oncology. In contrast to rigid image registration (RIR), which is employed at all stages of diagnosis and treatment, the uptake of DIR has been constrained by concerns over the potential for unsafe use. The AAPM Task Group 132 (TG132) published a report on the use of image registration, including many recommendations on clinical integration of registration in treatment planning and delivery. There is a remaining uncertainty on incorporating registration uncertainties into treatment margins (Sect. 6.A, TG 132), a challenge in clinical practice. The aim of this work was to report our experience in implementing a practical, patient specific quality assurance process based on the AAPM Task Group 132 report recommendations. This work includes refining our process of target contouring using PET with deformable image registration based on our experience of addressing vulnerabilities identified during implementation. A multidisciplinary team created a flowchart for patient specific quality assurance for image registration (RIR or DIR) based on use cases defined in the AAPM TG132 Report on the use of image registration in radiotherapy. Vulnerabilities identified from this implementation were assessed relative to AAPM TG132 recommendations. These findings were used to adapt our patient specific quality assurance to mitigate vulnerabilities. The main vulnerabilities were identified in the last steps of image registration. There was potential for inappropriate use of the registration for clinical use, such as target contouring where the image registration accuracy level was poor. Vulnerabilities were addressed by an adaptation in our quality assurance process. A new physics image registration QA task was introduced that independently checks registration accuracy and appropriateness of target contouring, addressing the vulnerability in the last steps of the AAPM TG132 flowchart. A multi-disciplinary team implemented the image registration process outlined by AAPM TG132. An improved patient specific quality assurance process was developed by introducing an independent physics image registration review that considers the acceptable registration uncertainty for the specific clinical use case in question.

**Supplementary Information:**

The online version contains supplementary material available at 10.1007/s13246-025-01541-1.

## Introduction

The use of imaging in radiation oncology is critical for diagnosis, target contouring, adaptive radiotherapy, and response assessment. While image modality and registration techniques may vary between departments, survey data show that image-guided radiotherapy is currently standard practice in the USA [1], Europe [2], and Australia [3]. There is a trend toward increasing complexity from simple anatomical matching to quantitative analysis of anatomical change [4] and from offline review to real-time adaptive radiotherapy [5, 6]. Challenges associated with these changes need to be addressed.

Rigid image registration (RIR) is limited, not accounting for motion within the body [7]. Deformable image registration (DIR) enables corrections for nonrigid variation in anatomy and assessment of the associated change in delivered dose from planned dose distributions [8, 9]. Multi-institutional accuracy studies performed across a range of anatomical sites [10–13] have shown that DIR is potentially superior to RIR in reducing registration errors. Residual inaccuracy in DIR can vary spatially across the image [13]. Due to the limitations of both DIR and RIR, multiple factors influence how departments choose to use DIR or RIR including clinical use case, anatomical site, or other details [14, 15].

In 2017, the AAPM Task Group 132 (TG132) published a report on the use of image registration. The report summarises techniques for RIR and DIR and describes the clinical integration of registration in treatment planning and delivery, providing many recommendations [16]. In particular, the report recommends assessment of registration accuracy levels (e.g., level 1 if the local region is aligned). These accuracy levels should then be communicated to the user of the registration and residual errors (registration inaccuracies) that cannot be resolved at final clinical use should be accounted for. There is a remaining uncertainty on how these registration uncertainties are incorporated into treatment margins, noted in TG132 as a lack of consensus in the radiation oncology community (Sect. 6.A, TG132).

This study aimed to: (a) develop a practical quality assurance process for deformable image registration of PET for target contouring, following AAPM TG132 recommendations; and (b) refine this process based on departmental experience, focusing on examining how registration uncertainties impact target contours and treatment.

## Methods

Implementation of the patient specific quality assurance for image registration was carried out by a multidisciplinary team consisting of three medical physicists, one radiation oncologist (RO), and one radiation therapist (RT). The scope of this work was to focus on a particular use case of image registration: target contouring on the planning CT based on visualization of PET with deformable image registration. It was noted that this registration is based on a CT-to-CT image registration (PET-CT with planning CT).

The team conducted this project by (i) Mapping out a flowchart of the initial quality assurance processes based on intended use and image registration techniques; (ii) assessing departmental experience of key vulnerabilities and issues; and (iii) formulating an adapted quality assurance process focused on addressing vulnerabilities and issues identified.

### Implementing a patient specific quality assurance process for use of image registration as per AAPM TG132

We implemented a patient specific quality assurance process as per AAPM TG132 “image registration report” (Appendix B of AAPM TG 132 “Example templates of an image registration request and report”) [16]. This process included performing data integrity and import checks (as per TG53 [17] and TG66 [18]) and contour assessment (Table IV in in AAPM TG132 [16]).

We created a flowchart for quality assurance processes for image registration, starting from (i) *upstream processes*, such as image acquisition, where the outcome from these processes affects the characteristics of the input image for the registration processes; followed by (ii) *image registration processes* which include the registration, assessment, as well as the communication of registration quality; and finally (iii) *downstream processes* which produce a clinically relevant object (e.g., GTV) by using the registered images to fulfil the intended use (e.g., target contouring while visualizing a resampled PET avidity map with deformable image registration). A detailed process for how image registration of PET is performed is provided in Supplementary 1, Sect. 1.

### Assessing vulnerabilities in the patient specific image registration QA process

The team assessed the departmental experience of the AAPM TG132 patient specific quality assurance map. Our combined experience was used to assess gaps or vulnerabilities in the processes for target contouring based on PET deformable image registration after implementation of AAPM TG132 for patient specific QA. This assessment was undertaken based on our departmental clinical systems: Velocity™ (Varian Medical Systems Inc., CA, US) for consolidation of imaging information and advanced image registration with DIR, Eclipse (Varian Medical Systems Inc., CA, US) for external beam radiotherapy treatment planning, Aria^®^ (Varian Medical Systems Inc., CA, US) oncology information system for post-treatment image assessment, approval, and storage of image registration report forms (image registration quality assurance results), and Truebeam^®^ linear accelerator console software (Varian Medical Systems Inc., CA, US) for treatment imaging.

### Adapting a patient specific quality assurance process for use of image registration based on the identified vulnerabilities

We adapted the patient specific quality assurance processes to mitigate issues found. The team considered the workflow in the department, considering how the processes flowed and were inter-connected. It was also noted that in our department the most appropriate registration depends on the registration accuracy achieved (e.g., DIR used when DIR accuracy is accurate and superior to RIR; RIR used when DIR accuracy is poor or inappropriate, such as when the patient’s arms are up vs. arms down).

The adapted quality assurance process to mitigate gaps aimed to ensure risk levels were acceptable to the organization [19] and within resource constraints [19, 20] while maintaining department efficiency.

## Results

### Implementing a patient specific quality assurance process for use of image registration as per TG132

**Fig. 1:**
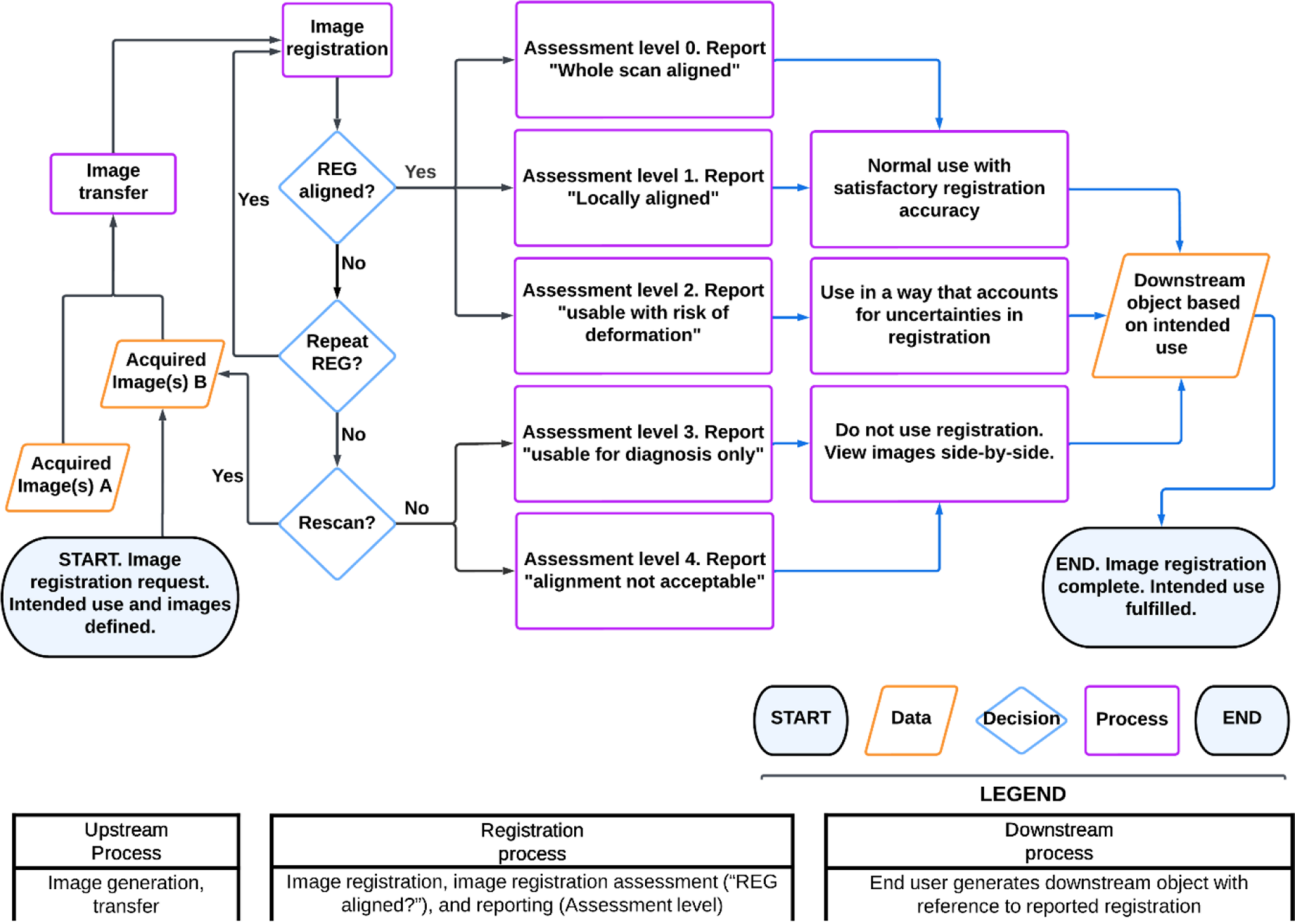
A flowchart for the implementation of patient specific QA of TG132, starting with an image registration request and ending with the image registration completed and intended use fulfilled. Note that “REG” refers to “image registration”; legend describes the icons for “start/end”, “data”, “decision”, and “process”; black arrows denote processes for the user performing image registration (e.g., RO), blue arrow denotes processes for the user who uses the image registration (e.g., RO); the assessment levels in this diagram are based on AAPM TG132^16^

We implemented the AAPM TG132 patient specific quality assurance processes for target contouring based on visualization of PET with deformable image registration. Figure 1 demonstrates this process showing flow and interlinking of components. The process *starts* with the images (“Acquired image(s) A” in Fig. 1) to be registered (“Image registration” in Fig. 1), which then undergo image transfer. In the image transfer process users import the data and may need to merge patient data when there are multiple medical record numbers (MRNs) for a single patient. The user then performs image registration and then qualitative image registration alignment checks (“REG aligned?” in Fig. 1) to assess the uncertainty assessment level (“Assessment level” in Fig. 1) as per AAPM TG132^16^. These steps are performed by Radiation Therapists (black arrows in Fig. 1) in the local department.

The accuracy of image registration alignment determines further paths: If the registration is aligned globally (”Assessment level 0” in Fig. 1) or locally (“Assessment level 1” in Fig. 1), there will be a report by the staff who registered the image (Radiation Therapist, RT) on registration accuracy (“Report “whole scan aligned”” in Fig. 1), to the user of the registration ( Radiation Oncologist, RO). The RO will perform a clinical process based on this (e.g., “Normal use with satisfactory registration accuracy” in Fig. 1); with these steps (such as target contouring) noted with blue arrows in Fig. 1. If the registration is not aligned, there may be a decision to try and address this by repeating registration (“Repeat REG” in Fig. 1) or performing a rescan. If a decision is made to use a registration with limited accuracy, the RT will assess the registration as useable for diagnosis only (“Assessment level 3” in Fig. 1). If the RT assesses the registration as unacceptable (“Assessment level 4” in Fig. 1), the user should then utilize the images without fusion (“Do not use registration. View images side-by-side” in Fig. 1). Decisions to rescan or reregister in the process map illustrate how paths can branch and emphasize that the clinical use of co-registered images should consider the registration accuracy, e.g., using a fusion view when local registration accuracy is satisfactory or a side-by-side view when registration accuracy is poor or marginal. The process ends when the downstream object is generated, where the image registration (and QA) is completed, and the intended use of the registration is fulfilled.

### Assessing vulnerabilities in the patient specific image registration QA process

Table 1 presents the vulnerabilities identified, assessed, and addressed in the image registration process in the department for target contouring based on visualization of PET. Column 1 in Table 1 lists the major processes identified. The team identified key vulnerabilities after implementation (more than 100 checks over 1 year) of patient specific quality assurance for image registration for target contouring based on visualization of PET, and these are described in column 2 of Table 1. Column 3 of Table 1 assesses these vulnerabilities with respect to AAPM TG132 or the department system software. These insights into the cause can be used to help find solutions to these vulnerabilities. The vulnerabilities were found to occur due to various factors including (a) scenarios that AAPM TG132 recommendations do not cover explicitly, (b) staff adherence to AAPM TG132 recommendations or (c) limitations of user interactions with our department software system. Column 4 of Table 1 notes how our study has addressed these vulnerabilities, which is further described in the subsequent section in the manuscript (Section C).

**Table 1:** Vulnerabilities identified, assessed, and addressed in the image registration process in the department for target contouring based on visualization of PET; U denotes upstream processes, R denotes registration processes, and D denotes downstream processes

Major process	Description of vulnerability identified after implementation of AAPM TG132 patient specific quality assurance	Assessing vulnerability with reference to AAPM TG132 or departmental system (Velocity)	Addressing Vulnerabilities of AAPM TG132 or departmental system
U1	Suboptimal frame of reference registration of PET vs. PET-CT: For tumors with motion, the PET avidity map is acquired over a long time (capturing the motion blur over multiple breathing cycles) whereas the PET-CT is acquired over a relatively fast pitch (capturing the image over typically one breathing cycle). This can result in differences in the position and size of a target defined by the PET.	AAPM TG132 partially addressed: does not explicitly state requirements for checking images that are registered with a frame of reference (FOR) registration Velocity partially addressed: a workflow is available that prompts the user to verify that the PET is registered to the PET-CT, corresponding to patient specific quality control guidelines for PET/CT for radiotherapy [21].	Additional physics patient specific check implemented to ensure FOR registrations are accurate; training and support to correct with new rigid. Gated PET/CT may be beneficial.
U2	Lack of detail in the image registration request: This can introduce delays in the image registration process such as transfer or image registration of the incorrect image type (PET TOF [22], NAC, or AC [23]), incorrect date (e.g. pre-chemotherapy vs. post-chemotherapy scans), or incorrect patient.	AAPM TG132 addressed: users of the image registration (RO) should specify the images to be used for image registration, or document this in standard protocols.	Additional physics check to independently check correct image transferred and registered.
R1	Limited registration accuracy due to sub optimal registration: For example, incorrectly registering T9 instead of T10, or use of an implausible image registration with negative Jacobian.	AAPM TG132 addressed: commissioning of rigid and deformable image registration algorithms with accounting for limitations of algorithms; patient specific awareness of the local regions of importance followed by using an appropriate registration technique.	Additional physics patient specific check of deformable image registration guided by a Velocity workflow.
R2	Obscurity over which registration file was clinically used: In challenging cases, the user may attempt to apply multiple or iterative rigid or deformable image registrations. There may be uncertainty on which is the final registration used when there are multiple registration files.	AAPM TG132 addressed: the image registration request should detail the image registration technique, with notes or comment on when multiple or iterative registrations are applied. Velocity partially addressed: all registrations and images have timestamps. However, registration filenames are not unique.	Additional physics check utilizing timestamps and unique identifiers in the case of multiple existing registrations
R3	Unclear definition of intended use or regions of interest: Image registration assessment of image registration accuracy levels may be limited if the intended use is not exactly specified. In some cases, some regions of PET avidity are not local areas of interest for treatment.	AAPM TG132 partially addressed: the image registration request should detail the regions for planning, however there are cases where there is uncertainty about which regions of PET avidity are to be treated and therefore require highest levels of image registration accuracy.	Additional physics check after RO target contour, integrated into a Velocity report to detail local area in the image registration accuracy check.
D1	Unclear how margins account for registration uncertainty: There is a lack of consensus advice about how image registration uncertainty (e.g. errors in image registration that cannot be resolved) should be incorporated into margins for GTV for target contouring based on PET visualization	AAPM TG132 partially addressed: the image registration report provides RO with uncertainties in the PET image registration. RO should assess accuracy level as well as the proximity of uncertainty to GTV.	Additional physics check to assess image registration accuracy level for PET with reference to appropriateness of RO PTV, CTV, or use of margins relative to uncertainty.
D2	Registration accuracy achieved not accounted for in contouring with PET visualization The end user of the image registration may miss the image registration report and miss image registration uncertainties.	AAPM TG132 partially addressed: the RO should assess and approve image registration as per plans, however there is a lack of a double check where this is missed. E.g., If the accuracy level achieved was 3 (poor) but RO assumed it was 1, then GTV contoured on PET avidity would lead to a geographical miss.	Additional physics check of the match of GTV vs. PET avidity with respect to the accuracy level achieved.

### Adapting a patient-specific quality assurance process for use of image registration based on the identified vulnerabilities

**Fig. 2:**
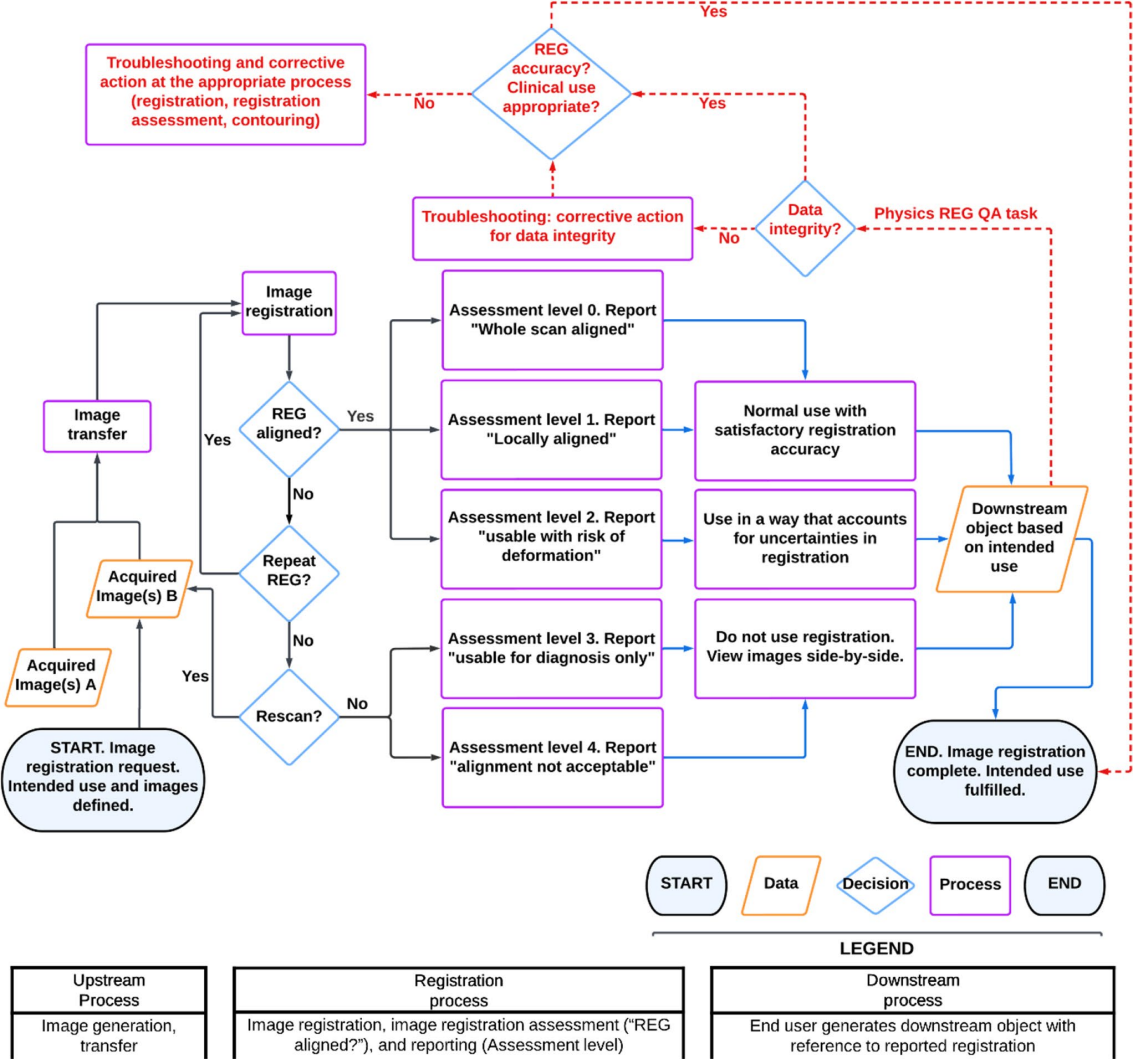
A modified process map with dotted red lines denoting a departmental offline physics image registration QA based on comparing the downstream object to registration starting with (i) an image registration request; (ii) upstream process of image generation, transfer, data processing; (iii) registration processes with registration, QA, and reporting; (iv) downstream processes where the end user generates the downstream object with reference to reported registration; and ending with (v) the image registration completed and intended use fulfilled. Note that “REG” refers to “image registration”; legend describes the icons for “start/end”, “data”, “decision”, and “process”; black arrows denote processes for the user performing image registration (e.g., RO), blue arrow denotes processes for the user who uses the image registration (e.g., RO), red arrows and text denote physics independent QA processes; dotted lines indicate these are done in parallel with clinical processes (which are not changed except for corrective actions of issues identified)

The action plan (column 4 of Table 1) addressed vulnerabilities identified (column 3 of Table 1) by implementing a new physics verification check in ARIA (“Physics REG QA task”) after target contours (downstream objects in Fig. 2) are generated, and before treatment planning commences. This “Physics REG QA task” is shown as red in Fig. 2.

Table 2 describes the new “Physics REG QA task” task for verification of image registration accuracy as well as target contouring based on PET visualization and how to implement this with a Velocity DIR QA workflow that includes a Jacobian check to detect unfeasible registration regions.

**Table 2:** The “physics image registration QA” for verifying target contouring based on PET visualization described on (a) a general non-vendor basis and (b) implemented in vendor-specific software (Velocity 4.1); more details on the velocity specific workflow is in sect. 1 of supplementary 1; more details on troubleshooting is in sect. 4 of supplementary 1

	NON-VENDOR SPECIFIC	VENDOR SPECIFIC SOFTWARE (Velocity 4.1)
Data integrity check	• Physics identify clinically relevant images, patients, registrations, and associated contours/doses, and ensure they are appropriately and clearly named. Assess image integrity such as geometry, orientation, warping.	• Physics identify clinically relevant images, patients, registrations, and associated contours/doses, and ensure they are appropriately and clearly named. Assess image integrity such as geometry, orientation, warping.
“REG accuracy?” (red text)	• Physics independently assesses the registration accuracy qualitatively, including PET-CT FOR registration (“Reg accuracy?” in red text in Fig. 2). If the registration is DIR, there is a quantitative assessment of DIR that includes a check to detect negative Jacobian values.	• Physicist runs Velocity 4.1 workflow “Review Deformable Registrations” is run. Note this is a standard workflow that comes with the clinical Velocity 4.1 software. • Primary image, Secondary image, deformable image registration, and contour of interest is selected (contour of interest representing target region, e.g. the clinical output) • The velocity workflow includes quantitative assessment, e.g., Jacobian checks (negative Jacobians indicate erroneous physical modelling as per 4.C.3 of AAPM TG132) • The velocity workflow produces a report which the user can add information to (text and screenshots) • The user performs qualitative assessment in Velocity software and adds screenshots to the Velocity report. This includes checking DIR accuracy level (as per Table VII in AAPM TG132).
“Clinical use appropriate?” (red text)	• Clinical assessment of downstream objects with registration accuracy ensures reasonable error handling in clinical use, particularly in regions of registration uncertainty. For example, If there’s a problematic match between a GTV_PET contour and misaligned PET avidity, a request for the RO to reassess the clinical GTV with available imaging (CT/MRI/PET) is appropriate.	• The user checks appropriateness of clinical target contours (GTV/CTV/PTV) as compared with propagation of SUV thresholds-based contouring to planning CT (if registration is locally accurate, SUV threshold-based contours should match GTV/CTV; if registration is inaccurate, these contours should not match GTV/CTV).
Troubleshooting and corrective action (red text)	• Communicate with relevant staff and resolve issue. • Document findings and actions taken towards resolving issues in an appropriate manner.	• The user copies the template text and then fills out the text report (see appendix for more information) • The user follows up on any correction actions noted, and uploads/exports the report (which includes DIR QA analysis, report text, and qualitative screenshots) to the oncology information system

## Discussion

The recommendations of image registration processes outlined in AAPM TG132 [16] have been used to develop safer image registration processes relevant to the risk profile in our department. In our study, a physics image registration check task was added after downstream use (Fig. 2) and before plan generation. This is complimentary to chart check suggestions outlined in AAPM TG275 [24] that are performed after the plan is finalized. Our approach directly assesses the appropriateness of clinical targets (CTV and PTV) against deformable image registration (DIR) uncertainty. Incorporation of this patient-specific quality assurance prior to treatment plan generation enables issues to be raised earlier in the process to efficiently execute corrections.

While our study aimed to show the flow and relationships of image registration for PET based target contouring, this is not likely to apply for other image registration use cases such as re-irradiation, dose propagation, and adaptive radiotherapy. Each specific use of DIR [16, 25, 26] requires unique consideration of processes and quality assurance, as well as how clinical responsibility of DIR is distributed. In Australasia, the specific role of the RO for target contouring and underlying image registration [27, 28] should be considered.

The work presented here may be of help to other clinics who wish to review their processes and associated risks, with the aim of reducing vulnerabilities in their own image registration workflows. Departments should develop internal guidelines that evaluate the uncertainties of image registration with respect to other residual uncertainties which include contouring, treatment delivery mode and respiratory motion [29]. For each image registration use case, guidelines should detail what to check with regard to image registration (Table 2 section a) and how to implement these checks within clinical software solutions (Table 2 section b).

To address the image registration vulnerabilities identified (U1, U2, R1, R2, R3, D1, D2 in Table 1), we evaluated quality management tools based on the effectiveness of preventative measures [20].

Automated interlocks or forcing functions were deemed impractical as there is a lack of automated patient specific DIR QA and associated ground truth (such as target contours) in commercial systems. We emphasized that our staff should use the Velocity PET registration workflow which prompts the user to check the registration accuracy of the frame of reference PET to PET-CT registration. If this is inaccurate (addressing U1 in Table 1) the RT can perform a more accurate rigid registration to override the frame of reference registration.

Simplification of the image registration workflow was deemed not possible due to image differences being patient dependent (as per Fig. 1) and deformable image registration being “ill-defined and over-constrained [16]”.

Verification and policies were considered feasible. Improved protocols were implemented to specify the PET imaging required for registration (addressing U2 in Table 1). Trained staff (physicist) were rostered to perform verification of the RT image registration and RO target contour based on PET visualization.

It is noted that as there is no automatic DIR QA in commercial systems, physicists are trained and responsible for quantitative DIR analysis. The type and timing of appropriate quality controls depends on the patient scenario and use case. Training is required to understand the limitations and uses of software systems associated with the deformable algorithms for patient-specific quality assurance. Clinical experience and an awareness of published validation studies is also beneficial.

There may be future work warranted for patient specific image registration quality assurance as practice patterns shift toward more complex use of image registration [14], such as iterative DIR [14]. It is not feasible to rely on quantitative QA as it is often not used during commissioning and is even less common for routine QA [15]. While there are efforts to use automated QA with deformable image registration metrics [30], such as an automated software tool to predict dose mapping accuracy [31], DIR still requires an individual human assessment informed by patient factors [32] such as site, disease characteristics and treatment intent. As per our findings in this study, automated reporting templates or rating tools can help with efficiency, and could be aligned with quantitative information [33]. Future work in this space could involve the integration of AI contouring to facilitate the optimization [34], or quality assurance QA checks [35] of DIR. There are promising opportunities to improve the safety and practicality of clinical DIR use with more advanced patient specific quality assurance.

While there is published guidance on deformable image registration by the AAPM TG132 report [16], more recent guidelines for DIR dose mapping [26], and DIR in clinical trials [36], remains challenging due to lack of ground truth and variability due to the wide range of use cases [37]. The quality management framework developed in this study could provide insight for other clinics to review their DIR QA structure and help mitigate image registration-related vulnerabilities.

## Conclusion

A multi-disciplinary team implemented a patient specific image registration process outlined by the AAPM TG132 for PET DIR for target contouring. We assessed vulnerabilities and enhanced the patient specific quality control process with the introduction of an independent physics image registration review. This review, performed after clinical target contouring, directly evaluates the appropriateness of the use of image registration based on the registration uncertainty achieved.

## Electronic supplementary material

Below is the link to the electronic supplementary material.

Supplementary Material 1
