# Supplementary material for: Improving patient specific quality assurance for image registration: clinical use case of target contouring for PET deformable image registration
Source: Phys Eng Sci Med. 2025 May 14;48(2):759–67. doi: 10.1007/s13246-025-01541-1 (PMC12209017; doi:10.1007/s13246-025-01541-1)
Supplement: Supplementary file 1 — Supplementary Material 1 [file 13246_2025_1541_MOESM1_ESM.docx]

# Supplementary 1: Example implementation of PET DIR QA

Section 1 has a detailed process for how image registration for PET is performed and then used clinically

The manuscript has a description of the process for physics PET DIR QA that checks registration accuracy with clinical output (target contour) in Table 2.

Section 2 has the template text used in the workflow described for physics PET DIR QA.

Section 3 has screenshots of the report generated by Velocity for physics PET DIR QA.

Section 4 has a detailed a description of failure modes and troubleshooting actions from an independent physics QA of PET DIR

## Section 1 Detailed process for performing image registration for PET

Note that this describes a detailed process for a PET image registration with a 4DCT image. Work processes for 3DCT are equivalent but without motion management.

*Abbreviations: RT is used for radiation therapist. RO is used for radiation oncologist. RPM refers to Varian Real-time Position Management system. PACS refers to a picture archiving and communication system. RTPS refers to radiation treatment planning system. MRN refers to medical record number. RIR refers to rigid image registration. VOI refers to volume of interest.PCT refers to planning CT.*

1. Upstream
   1. RT assess imaging RO instructions on imaging and image registration
   2. RT setup patient with immobilisation devices
   3. RT set scan length and field of view for planning CT (PCT)
   4. RT monitor patient breathing to setup 4DCT parameters (such as pitch)
   5. RT provide instructions for patient for regular breathing and acquire scan
   6. RT set breathing peaks on RPM waveform
   7. RT export RPM waveform to planning CT
   8. RT start 4DCT recon
   9. RT and/or Physicist review of 4DCT to check for artefacts, RPM amplitude, and appropriateness of pitch.
   10. RT/Physicist/RO review of findings of 4DCT check with appropriate actions (if applicable, e.g. repeat 4DCT)
   11. RT sends 4DCT to Eclipse RTPS
   12. RT imports 4DCT in Eclipse
   13. RT generates AVG image in 4DCT in Eclipse
   14. RT exports AVG image to Velocity (if free-breathing motion management technique; breath hold image to be sent for breath hold technique)
   15. RT uses PACS software to export PET imaging to Velocity
   16. RT imports images in Velocity and merges all images to correct MRN
2. Registration
   1. RT checks PET-CT with PET
   2. RT creates unique RIR and optimises PET-CT with PET (if required)
   3. RT runs Velocity workflow for PET to PCT registration
      1. Workflow confirms PET, PETCT and PCT
      2. RT manual rigid registration
      3. RT sets VOI and runs automatic rigid registration (optional)
      4. RT chooses DIR (optional) and runs DIR (optional)
      5. RT visually assesses PETCT-PCT registration accuracy (qualitatively)
   4. RT runs Velocity workflow for PET to PCT registration again (if required due to suboptimal registration)
      1. RT runs through all steps and then visually assesses PETCT-PCT registration accuracy (qualitatively)
   5. RT assess all registrations (if required)
      1. RT assesses registration accuracy of all relevant registrations
      2. RT decision on the registration is most appropriate (could be RIR or DIR) or if there are no images appropriate
      3. RT deletes registrations that are not used (if required)
   6. RT activates the clinical registration to be used if the PET is image accuracy level 0-2
      1. RT resamples PET
      2. RT exports resampled PET to Eclipse
   7. RT to assess actions if PET image accuracy level is 3-4 (not to be visualised registered)
      1. RT to export PET to Eclipse in different frame of reference (e.g. unregistered)
   8. RT repeats the above steps for each PET (e.g. pre-operative PET and post-operative PET)
   9. RT imports resampled PET in Eclipse
   10. RT documents image registration accuracy issues in ARIA (if required)
   11. RT finishes the image registration task in ARIA
3. Downstream
   1. RO reads task notes from RT in ARIA
   2. RO visualises PCT with resampled PET in Eclipse
      1. To visualise PCT with resampled PET in fusion mode if image accuracy level 0-2 (whole image aligned or locally aligned or aligned with uncertainties)
      2. To visualise PCT with PET in a side to side mode if image accuracy level 3-4 (not aligned or usable)
   3. RO generates GTV_PET with the assistance of resampled PET (if appropriate)

## Section 2: Template text that is used in DIR software report in Velocity

The template text that can be copied and pasted to the DIR software report is in the table below.

The user reviews the registration accuracy as well as checking the target contour is appropriate relative to the accuracy level achieved.

| PET DEFORMABLE IMAGE REGISTRATION PHYSICS QUALITY ASSURANCE  PRIMARY AND SECONDARY IMAGE USED:  PETCT and Planning CT (image name and date detailed in screenshots below)  INTENDED USE:  Target delineation with guidance from PET  REGISTRATION DETAILS:  Image name of PET file exported:  Registration file name linked to PET file exported:  Comment if multiple registration or PET files: (delete if none)  LOCAL REGIONS OF INTEREST:  Region of interest for QA defined by QA contour created with auto-segmentation -40% of SUVmax grown from the centre of tumour as visualised by RO target structures.  Comment (if any):  JACOBIAN SCREEN CHECK (Deformable)  Checked over the QA structure  Satisfactory: no negative Jacobian  Requires attention: negative Jacobian detected (comment)  REGISTRATION ACCURACY LEVEL (Deformable)  [delete lines that are not applicable][delete this section if deformable not used]  Accuracy level 0 achieved: whole scan aligned (within 2mm)  Accuracy level 1 achieved: locally aligned to tumour (within 2 mm)  Accuracy level 2 achieved: usable with risk of deformation  Accuracy level 2 recommendation: review whether PTV/PRV margins are required  Accuracy level 3 achieved: Usable for diagnosis only  Accuracy level 4 achieved: Alignment not useable  TARGET CONTOUR CHECK WITH registration QA (rigid or deformable)  [delete lines that are not applicable]  Accuracy Level 0-1 Pass: Target contours align on the fused PET image  Accuracy Level 0-1 Warrants attention: Target contours do not align on the fused PET image  Accuracy Level 2 Pass: Target contouring and margins are not required to be reviewed based on registration accuracy  Accuracy Level 2 Warrants attention: Target contouring and margins could be reviewed based on registration accuracy  Accuracy Level 3-4 Pass: Target contours not geometrically aligned to a fused PET, but reasonably aligned to corresponding anatomical  landmark.  Accuracy Level 3-4 Warrants attention: Target contours are geometrically aligned to a fused PET, but not aligned to corresponding  anatomical landmark  COMMENTS:  Staff performing image registration is by default performed by the RT completing the ARIA task.  Physicist checking resampled image:  This QA form was adapted by the Medical Physics Department from the AAPM TG132 QA report form (Brock, 2017, Medical Physics). |
| --- |

## Section 3: Example DIR software report snippets

This section includes what a completed Physics PET DIR QA looks like (without patient screenshot)


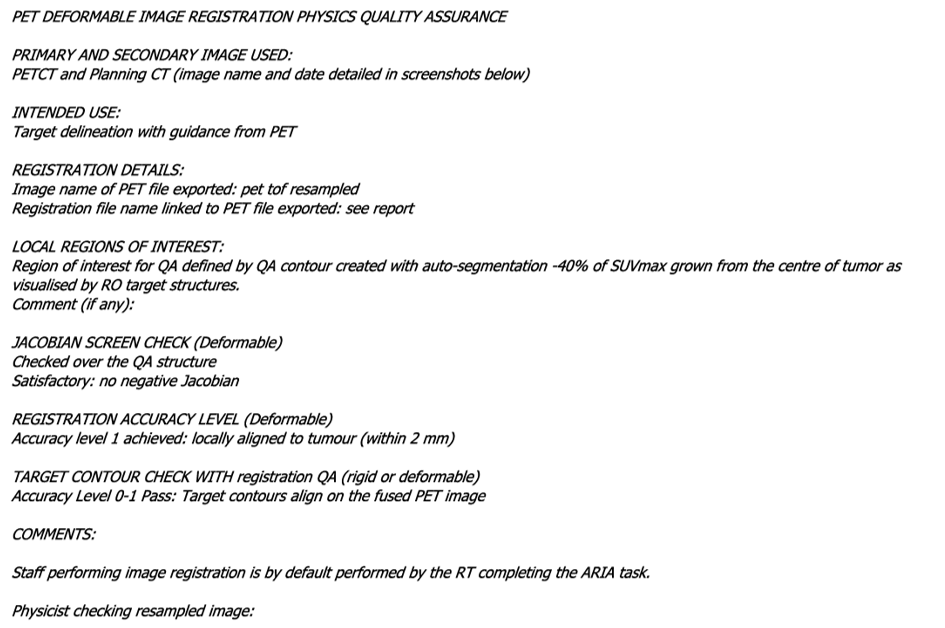


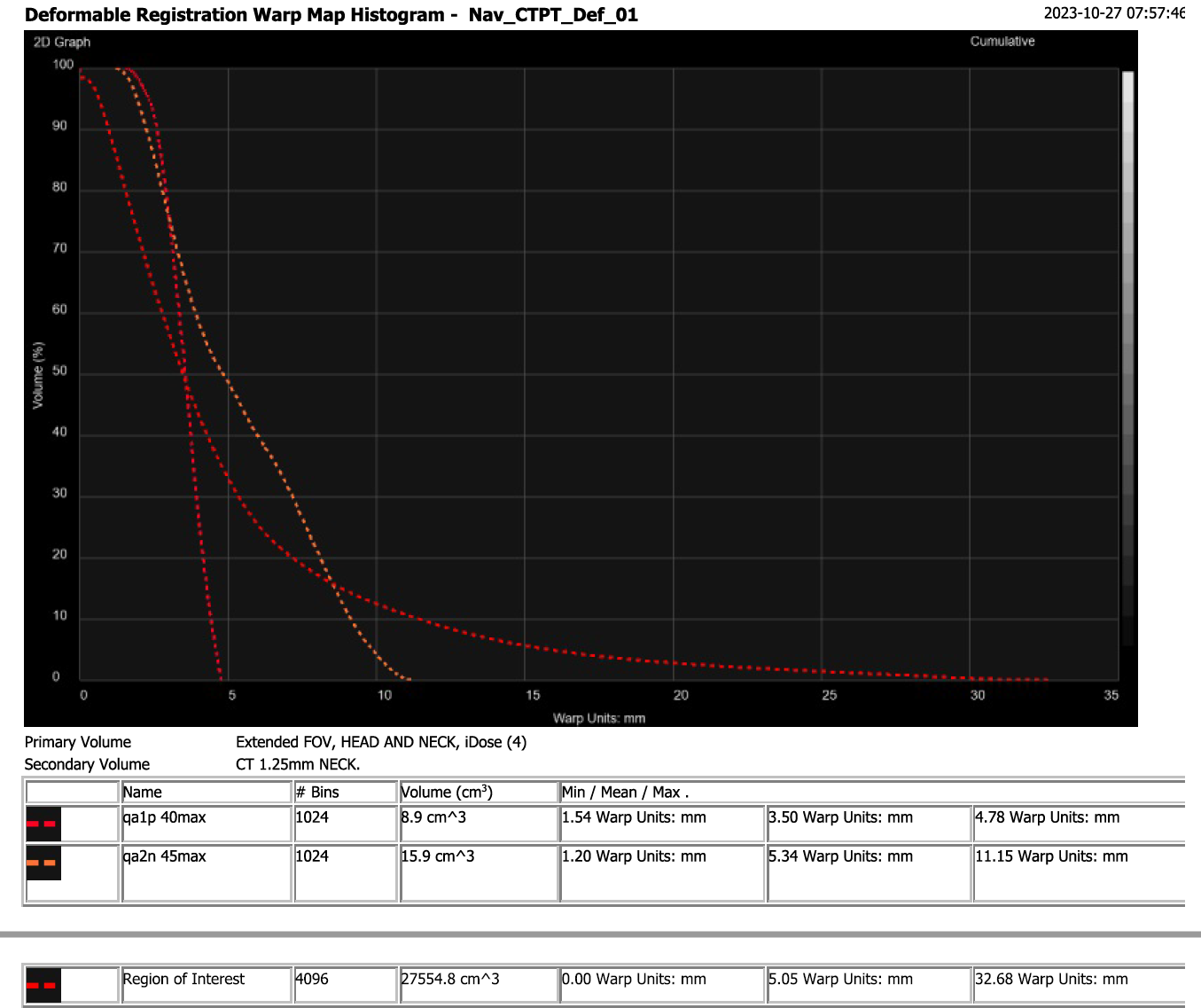

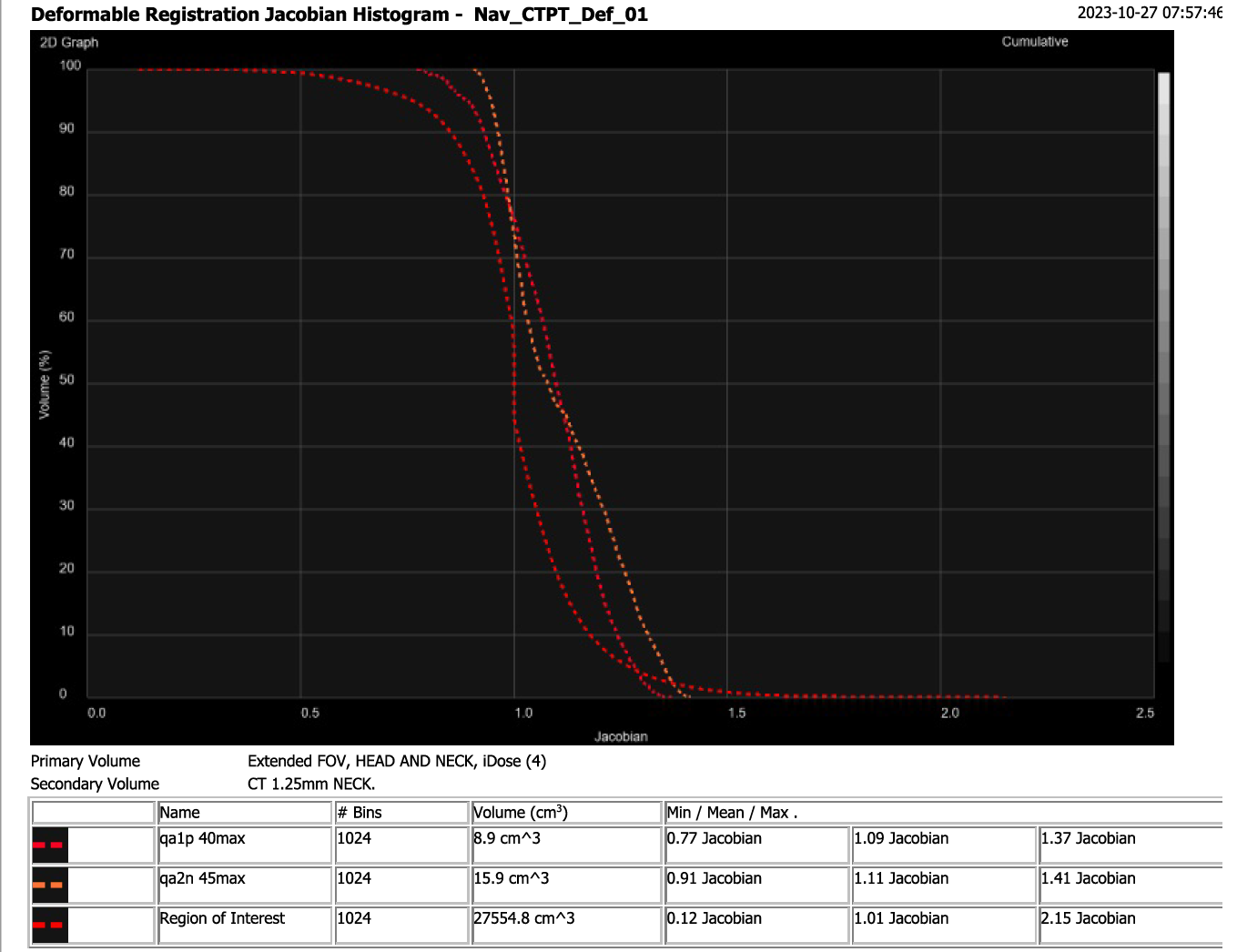


*Comment: screenshot of DIR propagated SUV threshold based contours compared against target contours (GTV/CTV/PTV) would be added after the Jacobian curve.*

## Section 4 Examples of troubleshooting and corrective action for physics DIR QA for PET

Note that this describes a detailed physics image registration QA based on a process for a PET image registration with a 4DCT image.

| **Data integrity check** | **Troubleshooting and corrective action** |
| --- | --- |
| Patient in Velocity has the incorrect MRN due to incorrect merging of images | Communicate to RTs.  Correctly merge to correct MRN |
| Unclear naming of multiple PET in Eclipse | Communicate to RTs and work towards clear naming of PET where there are multiple PETs. |
| Unclear use and status of multiple RIR and DIR in Velocity | Communicate to RTs that only clinical registrations should be in system. Retrospectively evaluate resampled files to work out which registrations were clinically used. |

| **Registration accuracy and clinical use appropriate check** | **Troubleshooting and corrective action** |
| --- | --- |
| PET SUV map is cut off where it is relevant to clinical target delineation; | Repeat RIR/DIR with larger VOI if appropriate to include larger area of PET avidity registration  Delete previous PET  Resampled and re-export improved PET |
| Inappropriate use of DIR when RIR would be optimal (such as with AVG PCT vs. AVG PET) or when DIR is misleading | Repeat with RIR  Delete previous PET  Resampled and re-export improved PET |
| Negative Jacobian in PET in region of interest | Repeat with RIR  Delete previous PET  Resampled and re-export improved PET  Communicate registration accuracy as well as notes on anatomical differences in images |
| There is a mismatch in the form of good registration accuracy and potential inappropriate registration use: e.g., PET DIR registration accuracy is good but GTV and GTV_PET does not follows PET avidity | Request RO to review GTV and GTV_PET with journal notes on any clinical decisions made (e.g. decision not to treat GTV due to patient history) |
| There is a mismatch in the form of poor registration accuracy and potential inappropriate registration use: e.g., PET DIR registration accuracy is poor, and GTV and GTV_PET follows PET avidity (which it shouldn’t be due to registration accuracy) | Assess DIR registration accuracy (with PETCT-PCT)  Assess registration accuracy notes in ARIA  Request RO to review GTV to not rely on GTV_PET geometrically (but can use PET as approximate areas of avidity) |
